# From tissue to silicon to plastic: three-dimensional printing in comparative anatomy and physiology

**DOI:** 10.1098/rsos.150643

**Published:** 2016-03-02

**Authors:** Henrik Lauridsen, Kasper Hansen, Mathias Ørum Nørgård, Tobias Wang, Michael Pedersen

**Affiliations:** 1 Comparative Medicine Laboratory, Department of Clinical Medicine, Aarhus University, Aarhus, Denmark; 2 Zoophysiology, Department of Bioscience, Aarhus University, Aarhus, Denmark; 3 Aarhus School of Architecture, Aarhus, Denmark

**Keywords:** three-dimensional printing, additive manufacturing, comparative anatomy, comparative physiology, imaging

## Abstract

Comparative anatomy and physiology are disciplines related to structures and mechanisms in three-dimensional (3D) space. For the past centuries, scientific reports in these fields have relied on written descriptions and two-dimensional (2D) illustrations, but in recent years 3D virtual modelling has entered the scene. However, comprehending complex anatomical structures is hampered by reproduction on flat inherently 2D screens. One way to circumvent this problem is in the production of 3D-printed scale models. We have applied computed tomography and magnetic resonance imaging to produce digital models of animal anatomy well suited to be printed on low-cost 3D printers. In this communication, we report how to apply such technology in comparative anatomy and physiology to aid discovery, description, comprehension and communication, and we seek to inspire fellow researchers in these fields to embrace this emerging technology.

## 1. Background

Comparative anatomy and physiology, the study of structural and functional similarities and differences within the animal kingdom, are intrinsically three-dimensional (3D) disciplines. However, while anatomy and many macroscopic physiological processes, such as blood displacement, phenotypic flexibility of organs and muscle movement are best understood in 3D space, written descriptions of structures and functions have traditionally been restricted to two-dimensional (2D) figures. Since the Renaissance, naturalists and physicians have painstakingly dissected and vivisected animal as well as human subjects and documented their findings in drawings and later with photography. This effort laid the foundation for the current knowledge in comparative anatomy and physiology, and although careful dissection is probably the most thorough method of acquiring structural information, this can be extremely time consuming and discoveries are intrinsically hard to communicate in text or as 2D figures. Fortunately, recent advances in computational power, imaging software and 3D-imaging systems now allow for the construction of videos and interactive models from images obtained by modern imaging technologies, such as X-ray computed tomography (CT), magnetic resonance imaging (MRI) and optical projection tomography (OPT) [1–4]. Such 3D virtual models and accompanying animations are extremely valuable to visualize complex anatomical structures [5], and can now be viewed as a standard when reporting novel anatomical and physiological findings. However, these virtual models remain hampered by the necessity to project the models on 2D screens. This limitation can be alleviated by the recent access to rapid prototyping and additive manufacturing technologies. Although representing a technology several decades old [6], it is only recently that additive manufacturing, or 3D printing in a more recently popularized terminology, has become affordable even for private persons through the introduction of simple, low-cost desktop 3D printers [7].

Medical researchers realized early on the potential of 3D printing to visualize complicated pathological conditions and as a method to design and produce tailored implants and prosthetic devices, but also as a way to produce supplementary teaching resources for anatomical training traditionally curtailed by the low accessibility of donated bodies [8–10]. Recently, veterinary medicine has started adopting 3D print technology for similar uses in well-known species [11,12].

Anatomical structures can be very complex in different species, and while interactive virtual *in silico*3D models have been used in comparative anatomy and physiology, the next step of generating physical scale models ‘*in plastico*’ remains to be fully exploited. Websites such as DigiMorph (http://www.digimorph.org/), Smithsonian X3D (http://3d.si.edu/) and Inside-Zoo (http://www.inside-zoo.com/) already host a number of downloadable 3D print files, but the vast potential of these models remains to be realized. It is our experience that most researchers and students prefer ‘seeing with their hands’, and we believe that generating large size manageable physical models can aid the discovery, description, comprehension and communication of anatomical and physiological features. This communication provides an introduction to 3D printing and a step-by-step demonstration of how to convert perishable tissue structures into imperishable scale models. Finally, we discuss the potential contribution of 3D printing technology in the field of comparative anatomy and physiology.

## 2. The steps from a biological sample to a three-dimensional printed model

**Figure 1.**
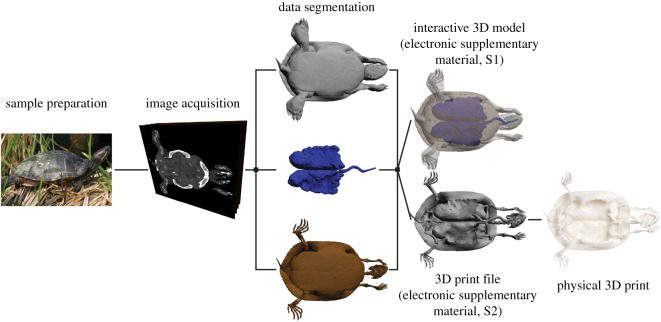
From tissue to silicon to plastic. The process of producing 3D prints of anatomical structures exemplified in a red-eared slider (*Trachemys scripta elegans*). Sample preparation initiates the process followed by image acquisition applying one or several types of imaging equipment, CT, MRI, PET, US, etc. Structures of interest are segmented digitally allowing for the production of interactive virtual 3D models and printed physical models. The interactive slider model is available as the electronic supplementary material, S1, and the ready-to-3D-print STL-file of the same model is available as the electronic supplementary material, S2.

**Figure 2.**
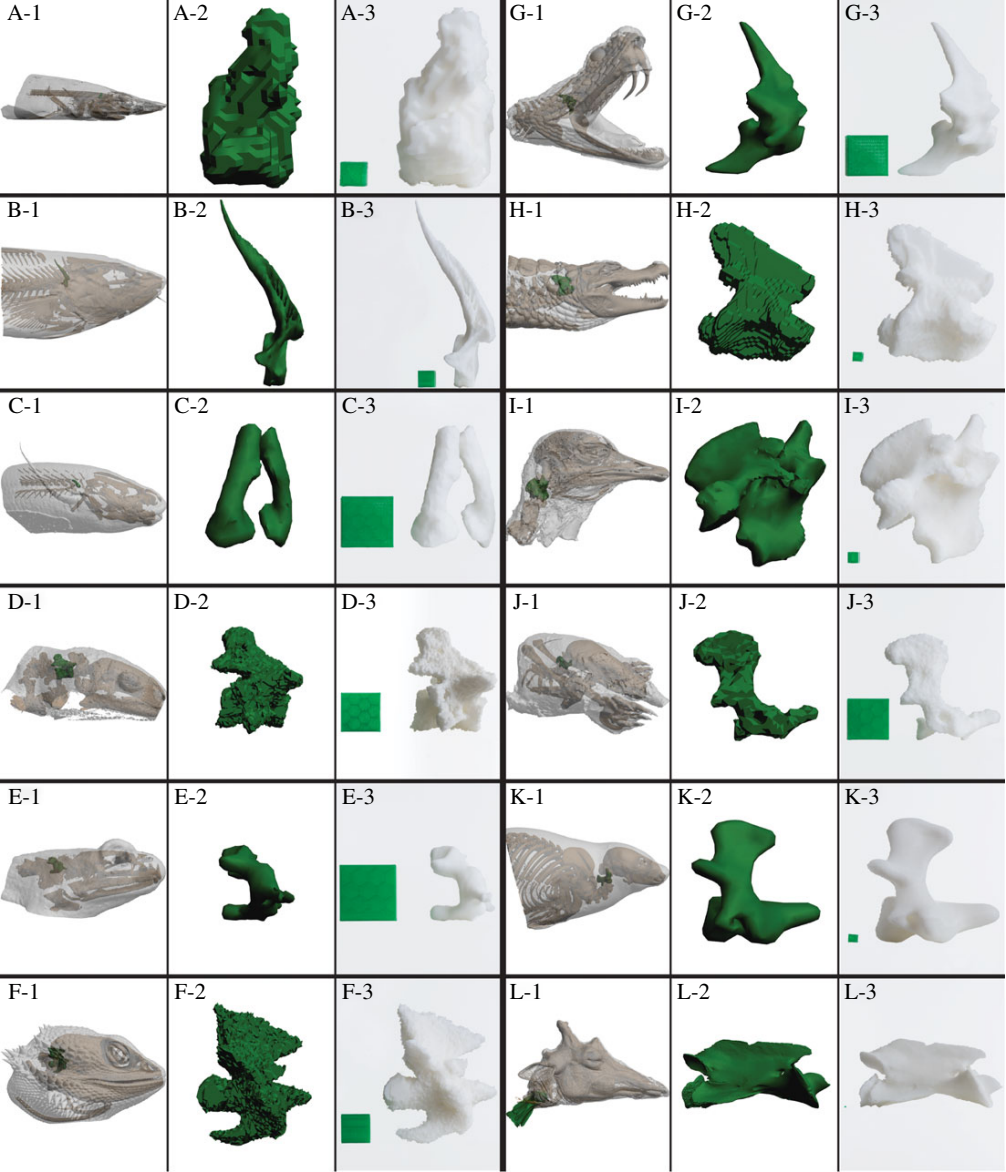
3D printing of corresponding structures in several species. The second vertebra, axis, of representative species of present gnathostome classes. The first column (1) for each species shows axis placement in the original sample, the second column (2) displays a digital axis model and the third column (3) displays a 3D printed version of the axis. (A-1 to A-3) school shark (*Galeorhinus galeus*), print scale: 250%; (B-1 to B-3) Atlantic cod (*Gadus morhua*), print scale: 400%; (C-1 to C-3) African lungfish (*Protopterus annectens.*), print scale: 1192%; (D-1 to D-3) tiger salamander (*Ambystoma tigrinum*), print scale: 1100%; (E-1 to E-3) European common frog (*Rana temporaria*), print scale: 1154%; (F-1 to F-3) bearded dragon (*Pogona* sp.), print scale: 600%; (G-1 to G-3) puff adder (*Bitis arietans*), print scale: 950%; (H-1 to H-3) black caiman (*Melanosuchus niger*), print scale: 150%; (I-1 to I-3) ostrich (*Struthio camelus*), print scale: 200%; (J-1 to J-3) European mole (*Talpa europaea*), print scale: 1000%; (K-1 to K-2) harbour seal (*Phoca vitulina*), print scale: 150%; (L-1 to L-3) giraffe (*Giraffa camelopardalis*), print scale: 50%. All models were scaled to appear as similar sized. Printed scale cubes (green) represent 2.5×2.5×2.5 mm^3^ for each model.

**Figure 3.**
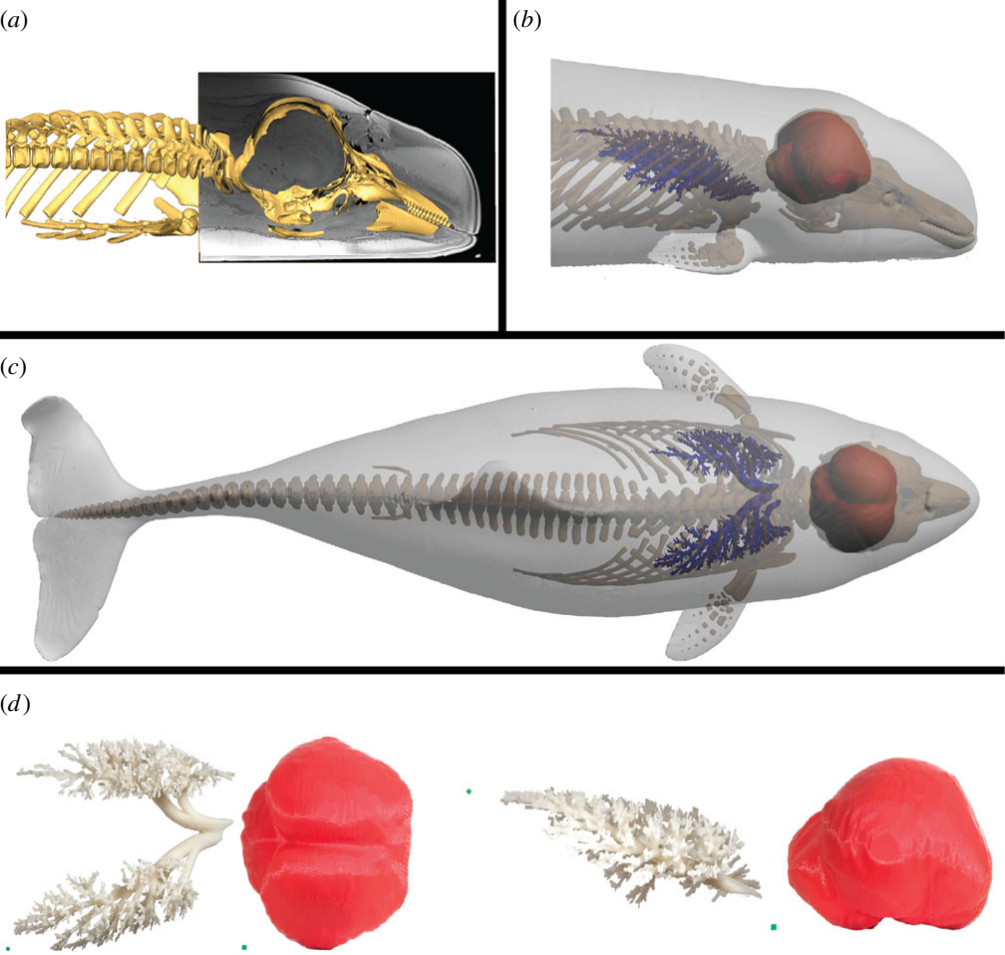
3D printing of negative spaces. Interior or negative spaces are non-dissectible but can easily be segmented from imaging data and prepared for 3D printing. The interior of the skull and air-filled lungs of a harbour porpoise (*Phocoena phocoena*). (*a*) Lateral view of the porpoise demonstrating MRI data registered to CT data, allowing for segmentation of the skull interior as demonstrated in (*b*). (*c*) Digital model of the skull interior and air-filled lungs. (*d*) 3D printed models of the skull interior and air-filled lungs, print scale: 80%. To produce the complex lung model, a material extrusion printer with a dual printer head was applied, which allowed for support material dissolution after printing. Printed scale cubes (green) in (*d*) represent 2.5×2.5×2.5 mm^3^ for each model.

**Figure 4.**
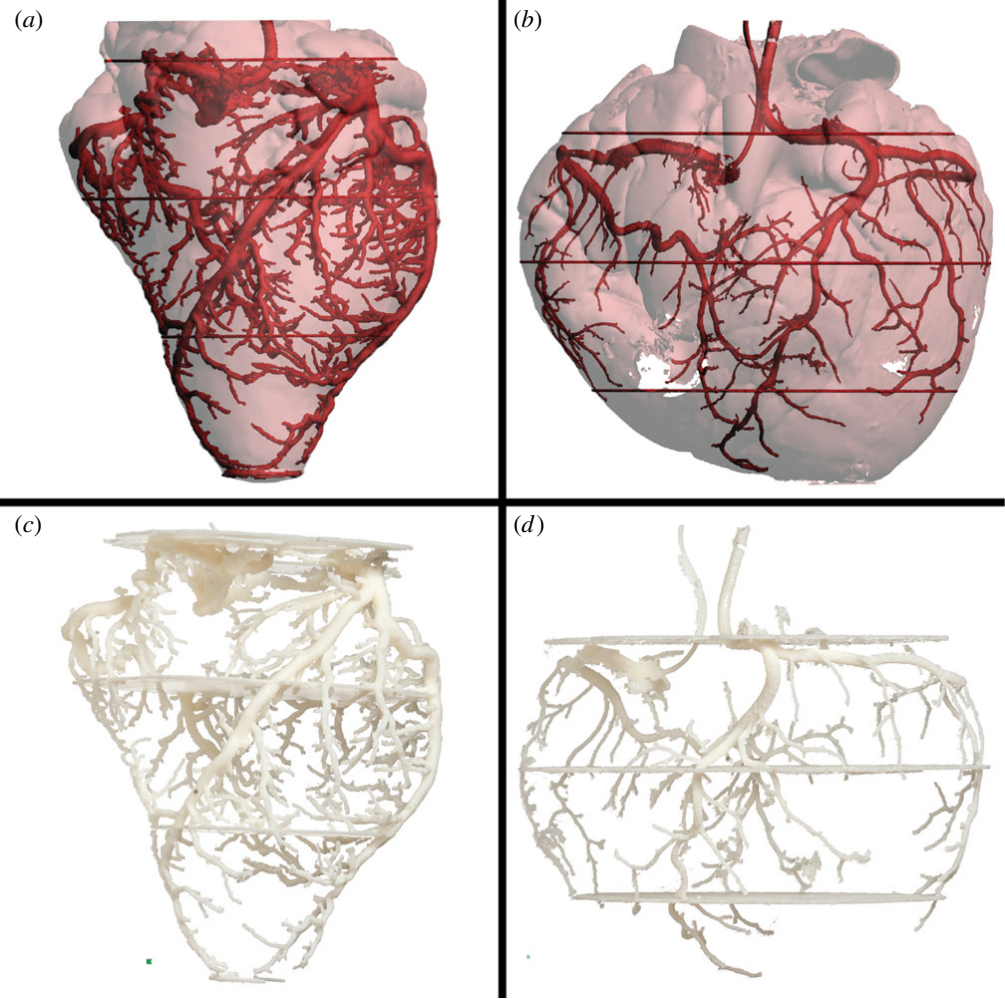
3D printing of complex anatomical structures. The coronary arterial mesh of the giraffe (*Giraffa camelopardalis*) (*a*,*c*) and African bush elephant (*Loxodonta africana*) (*b*,*d*) hearts. To support the delicate structures of the vasculature, four discs were added to the giraffe heart and three discs to the elephant heart in the 3D printed models. To produce these complex heart models, a material extrusion printer with a dual printer head was applied, which allowed for support material dissolution after printing. The giraffe heart (*c*) was scaled to 70% and the elephant heart (*d*) to 35% prior to printing. Printed scale cubes (green) in (*c*,*d*) represent 2.5×2.5×2.5 mm^3^ for each model.

**Figure 5.**
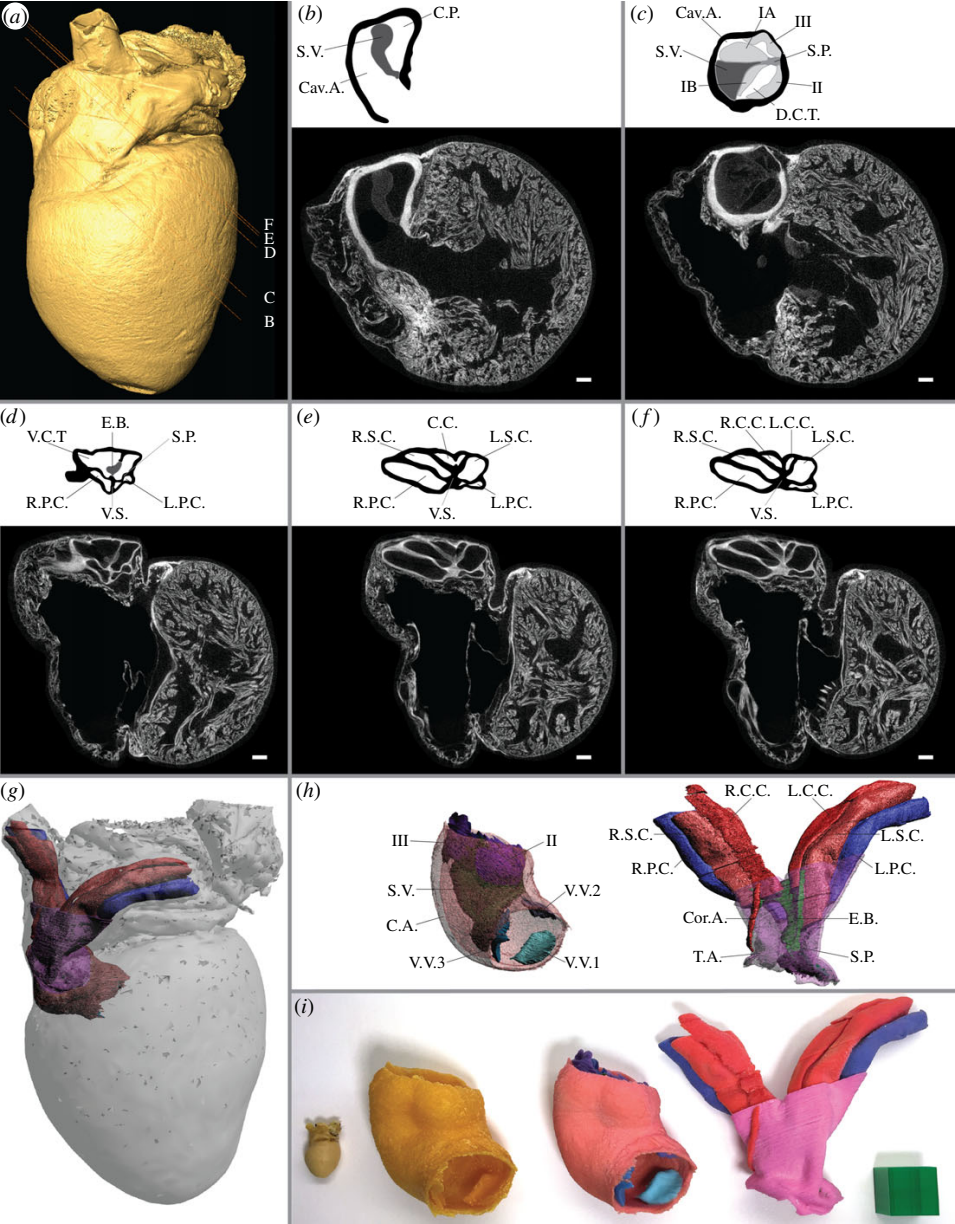
3D printing of complex anatomy in colour coded models. The anuran outflow tract, here exemplified in the cane toad (*R. marina*), is a highly complex anatomical structure consisting of a conus arteriosus where oxygenated and deoxygenated blood is functionally though not physically separated and a truncus arteriosus where the carotid, systemic, pulmonary and coronary arteries arise. (*a*–*f*) Sectional images generated using μCT imaging to represent similar sections as fig. 3 of [22]. Placement of sections is shown in (*a*). Scale bar is 1 mm. (*g*,*h*) Digital model of the complete toad heart (*g*) and the conus arteriosus and truncus arteriosus, respectively, left and right in (*h*). (*i*) the original toad heart specimen (left) compared to a monochromatic PLA plastic 3D print of the conus arteriosus (second from left), a multi-coloured sandstone 3D print of the conus arteriosus and all appertaining structures (third from left) and a multi-coloured sandstone 3D print of the truncus arteriosus and all appertaining structures (right). Print scale in all models is 1000%. Printed scale cube (green) in (*i*) represents 2.5×2.5×2.5 mm^3^. C.A. conus arteriosus; Cav.A. cavum aorticum; C.C. carotid canals; Cor.A. coronary artery; C.P. cavum pulmo-cutaneum; D.C.T. dorsal chamber of truncus; E.B. endothelial block in ventral chamber of truncus; L.C.C. left carotid canal; L.P.C. left pulmo-cutaneous canal; L.S.C. left systemic canal; R.C.C. right carotid canal; R.P.C. right pulmo-cutaneous canal; R.S.C. right systemic canal; S.P. septum principale; S.V. spiral valve; T.A. truncus arteriosus; V.C.T. ventral chamber of truncus; V.S. vertical septum. IA, IB, II and III, valves at anterior end of the bulbus arteriosus; V.V.1, V.V.2 and V.V.3, bulbo-ventricular valves.

**Figure 6.**
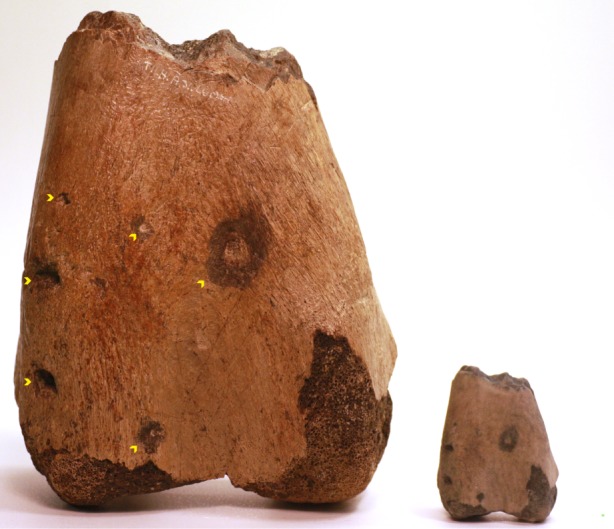
3D printing in full colour to replicate unique specimens. The fossilized lower portion of a hadrosaur (*Hadrosaurus* sp.) femur carrying bite traces (yellow chevrons) resulting from predation or scavenging. The original specimen was digitized using overlapping photography and printed as a 30% scale model in full colour sandstone. Printed scale cube (green) to the very right represents 2.5×2.5×2.5 mm^3^.

The process from having the biological specimen in hand to the final 3D print can be divided into four discrete steps: (i) sample preparation, (ii) image acquisition, (iii) data segmentation, and (iv) printing (figure 1). This process of generating anatomical 3D prints is exemplified for bone models (the axis bone of 12 different gnathostomes, figure 2), air-filled space of the lungs and the internal surface of the cranium in a harbour porpoise (i.e. negative surfaces, figure 3), complex structures such as the coronary vasculature of giraffe and elephant hearts (figure 4) and the outflow tract of the cane toad heart (figure 5), the fossilized lower femur of a hadrosaur replicated in true colours (figure 6), and finally a surface and bone reconstruction of the anterior section of a tiger salamander to demonstrate 3D printing in metal. Additionally, we provide an interactive PDF model (electronic supplementary material, S1) and a ready-to-3D-print file of a red-eared slider containing skeletal elements and lungs (electronic supplementary material, S2).

### 2.1 Step 1: sample preparation

Sample preparation is important and potentially the determining factor for a 3D print endeavour to succeed. Optimal sample preparation varies between different imaging modalities; therefore, subsequent image acquisition should be considered already at the sample preparation step. Calcified structures such as vertebrae (figure 2) are readily imaged using X-ray CT without any prior sample preparation other than anaesthesia for *in vivo* imaging or sample fixation for *post mortem* imaging. Even freezing results in very little modifications of solid tissue (calcified or keratinous tissue) and does not affect CT acquisition significantly, and can therefore be used when suitable. For soft tissue CT imaging, several non-specific heavy element staining techniques exist. By applying the passive diffusion of iodine or heavy metal ions such as mercury, silver or lead that will accumulate at different concentrations in disparate soft tissue compartments, it is possible to generate CT image contrast between these compartments that are usually indistinguishable with CT (figure 5) [13,14]. Furthermore, a technique for antigen-specific heavy metal staining to target specific gene products in a sample has been described [15]. Endocast production of vascular spaces, such as the coronary artery mesh of hearts (figure 4), can be achieved by perfusion with a fluid that solidifies shortly after injection and carries a contrast agent, such as salts of lead or barium for CT imaging or gadolinium for MRI.

### 2.2 Step 2: image acquisition

Imaging is a vast and rapidly expanding field within medical sciences and includes CT, MRI, OPT, 3D-ultrasound (3D-US), positron emission tomography (PET) and single-photon emission computed tomography (SPECT) [16]. Principally, any modality capable of generating digitalized 3D information of an organ or tissue is useful for the purpose of generating an anatomical 3D print; however, CT and MRI are most commonly used and are often the most applicable techniques. CT and MRI generate stacks of 2D images through the sample typically in DICOM (Digital Imaging and Communications in Medicine) or TIFF (Tagged Image File Format) format that can be analysed directly or rendered into 3D volume or surface visualizations. CT relies upon back projection reconstruction of several X-ray-generated projections, and most clinical CT equipment operate with an X-ray source voltage in the range of 80–140 keV, whereas high-resolution micro-CT (μCT) equipment usually employ lower voltages (25–80 keV). X-ray source voltage should be optimized for each specific sample, and as a rule of thumb, selecting a low voltage will enhance contrast between heavy element structures such as calcified tissue and contrast agents, whereas a higher voltage increases the signal-to-noise ratio [17]. X-ray CT generates ionizing radiation and radiation dose must be considered when performing CT on animals *in vivo*. CT is very suitable for calcified tissue reconstruction and segmentation, and by using the correct sample preparation such as heavy element soft tissue staining or vascular perfusion with an X-ray contrast agent, the applicability of CT for image acquisition can be expanded to cover a wide range of tissue visualizations.

MRI can generate images with distinct contrast between soft tissues. MRI equipment exists in large sample, low-resolution, low-field (1.5–3 Tesla) systems, and small sample, high-resolution, high-field (7–14 Tesla) systems. Generally, MRI systems are more expensive than CT systems and require more training. While specialized MRI sequences and protocols can be constructed for specific needs, it is our experience that basic MRI sequences may satisfy most needs for anatomical visualization.

2D image analysis does not require isotropic image acquisition, i.e. image resolution is not equal to slice thickness. This is often the case in more traditional biological imaging techniques such as multiple slice microscopy or *z*-stack generation using confocal microscopy in which slice thickness can exceed image resolution by several orders of magnitude. However, for generating 3D surfaces and volumes, it is generally desirable to acquire isotropic data allowing for oblique slice generation in the subsequent segmentation process. Additionally, image acquisitions should optimally be performed at a resolution equal to or higher than the print resolution of the 3D printer intended to produce the final plastic models in order not to lose accuracy [10]. This is exemplified in figure 2, where spatial data acquired in the cod, lungfish, frog, puff adder, harbour seal and giraffe is at a sufficient resolution to produce smooth and accurate physical scale models of the axis bone, whereas spatial data acquired in the shark, salamander, bearded dragon, caiman, ostrich and mole is at a lower resolution relative to animal size, only allowing for reproduction of the models with a reduced detail level.

Image acquisition is generally the most expensive step of generating 3D prints, and many comparative physiologists or anatomists may not have easy access to medical imaging equipment; however, this can be solved by forming collaborations with clinical departments when complying with simple hygienic precautions. Alternatively, commercial providers of imaging services are available.

3D surface models of physical structures can be obtained by a variety of commercial 3D scanners typically applying an infrared optical sensor or a touch probe. However, a very useful freeware to generate full colour and highly detailed surface models from a series of up to 70 overlapping photos acquired with a digital camera was recently made available from Autodesk 123D Catch (http://www.123dapp.com/). In a photogrammetry process that simply involves capturing overlapping photos from all sides of the object of interest and swift 3D rendering using cloud computing, Autodesk 123D Catch allows the user to produce full colour 3D surface models of millimetre as well as metre-sized objects. However, the option of visualizing underlying structures is unavailable with this surface visualization technique.

### 2.3 Step 3: data segmentation

Image acquisition is fundamentally passive, recording spatial information of the object under study without annotating any anatomical structures to certain organs or functions. Data segmentation, in contrary, is a manual (or semi-automatic) process of appointing recognizable structures to specific entities. Image segmentations can be made in freeware such as OsiriX Dicom Viewer (www.osirix-viewer.com) or ImageJ (http://imagej.nih.gov/ij/), both offering a wide range of segmentation and 3D rendering plugins. However, most studies in digital anatomy use Amira (http://www.vsg3d.com/amira/overview), a commercial 3D analysis software for advanced image segmentation [3,5,18–20].

Data segmentation is carried out manually or semi-automatically, drawing regions of interest in multiple 2D sections of the sample and assigning these regions to a volumetric structure. Ruthensteiner & Hess [3] provide a thorough description of how to segment and imbed 3D models in interactive PDF files [3]. Following image segmentation, a surface model is generated and exported in STL-format (STereoLithography or Standard Tessellation Language) for monochromatic models or alternatively VRML-format (Virtual Reality Modelling Language) or X3D-format (Extensible 3D Graphics) for models containing a coloured texture. Subsequently, the model can be rescaled in computer-aided design (CAD) software, e.g. the freeware Netfabb basic (http://www.netfabb.com/), and support material can be added to strengthen fragile structures (figure 4).

### 2.4 Step 4: printing

Several 3D printers are available on the market, applying a few different additive manufacturing technologies. The American Society for Testing and Materials (ASTM) has attempted to standardize the terms, nomenclature and acronyms associated with additive manufacturing technologies [21]. Though this standardization remains to become widely adopted in the additive manufacturing industry currently awash with different terms to describe similar technology, we will comply with the ASTM standards in the following. The ASTM categorizes current 3D printing technologies into seven categories: binder jetting, directed energy deposition, material extrusion, material jetting, powder bed fusion, sheet lamination and vat photopolymerization. At present, the majority of low-cost commercial 3D printers are of the material extrusion type (commonly known as fused deposition modelling) [11]. As these printers are affordable, popular and perform well in producing anatomical models, this technology will initially be discussed here. Material extrusion 3D printers can be compared to an automatic and motorized glue gun, extruding a cable of raw material through a movable printer head, heating and melting the material, and depositing it into a thread-like model. As the printer builds consecutive layers of material at a predefined path and an adjustable step size, the shapes of the model start to appear. To support fragile elements of the structure during printing, a thin, removable (in some cases dissolvable) layer of support material is laid around the structure. The final printed model is robust and self-supporting, and support material is removed by hand or using a pair of tweezers. Material extrusion printers are typically supplied by thermoplastics such as polylactic acid (PLA), acrylonitrile butadiene styrene (ABS), by rubber-based materials or even metal [11].

Relatively simple shapes without protrusions and fragile elements are optimal for material extrusion type 3D printing. Bone elements, such as the axis bone of 12 different vertebrates in figure 2, are examples of structures that are easily reproduced by material extrusion using thermoplastics. On the other hand, complex and delicate structures exemplified in this communication by a model of internal air-space in lungs (figure 3), and the coronary arterial mesh of hearts (figure 4), are structures that can pose some difficulties for material extrusion type printing as support material can be difficult to remove without damaging the intricate anatomical structures in these models. However, this is solved by using a dissolvable support material (figures 3 and 4).

A limiting factor of current material extrusion technology capable of extruding only one or a few materials simultaneously is the inability to produce full colour objects. A print of a complex anatomical structure containing several segments is often easier to comprehend when individual segments are colour coded, which is standard practice when producing digital interactive models [1,3–5]. To circumvent this limitation, binder jetting 3D print technology can be applied as this technique allows for full colour models. By successively depositing coloured binder material onto layers of powder, typically consisting of plastic or sandstone, binder jetting allows for the production of full colour anatomical and physiological models. To demonstrate the usefulness of this technique in reproducing highly complex anatomy, we examined the outflow tract of the cane toad (*Rhinella marina*) heart. The anatomy of the anuran outflow tract was previously described in detail in xenopus (*Xenopus laevis*) by de Graaf in 1957 [22], who provided impressive but almost incomprehensible cross-sectional drawings through the conus arteriosus and truncus arteriosus of the outflow tract. To investigate potential similarities and differences between de Graaf’s original findings in xenopus obtained by extensive dissections and our findings in the cane toad applying soft tissue staining and modern μCT, we produced a virtual model of the cane toad heart. In figure 5 and electronic supplementary material, S3, the anatomy of the outflow tract of the cane toad is demonstrated using a similar cross-sectional format as applied by de Graaf [22] (figure 5*a*–*f*) as well as using 3D virtual models (figure 5*g*,*h*) and finally as printed models in full colour sandstone (figure 5*i*). Even though the cross-sectional representations of the outflow tract contain information equal to physical 3D models, physical scale models make complex anatomy more accessible and have the potential to increase comprehension and communication of complex anatomical and physiological features.

The colours of the 3D printed models in figure 5 and electronic supplementary material, video S3 are artificially selected to distinguish separate segments of the models. However, replicating the true colours of an original coloured structure is also possible using full colour binder jetting. To demonstrate this, we prepared a 3D digital model of a fossilized hadrosaur (*Hadrosuarus* sp., Late Cretaceous) distal femur fragment carrying several bite traces [23]. By using 67 overlapping photos as described in the image acquisition section, we generated a 3D digital texture model of the fossil that was subsequently printed as a 33% scale model in full colour sandstone (figure 6 and electronic supplementary material, S4). The implications of this relatively straightforward approach of digitizing, e.g. fossils or rare type specimens in the collections of natural history museums and private collectors as well as archaeological findings would be the possibility to provide a potential research fellow on the other side of the planet access to print his/her own version of the specimen in full colour and at a desired scale, simply by attaching the print file to an e-mail rather than physically shipping the specimen [24]. Some investigations obviously require access to the original specimen; however, when examining surface properties or performing geometric measurements a printed model may suffice.

Binder jetting 3D printing can apply metal powders such as bronze, iron and stainless steel that are glued with a temporary binder solution which is later replaced with bronze by heating the printed metal powder object to 2000^°^C in a kiln containing liquefied bronze [7]. This procedure creates robust and durable metal models exemplified here in a bronze infused stainless steel 3D print of the anterior skeletal and surface anatomy of a tiger salamander (*Ambystoma tigrinum*; electronic supplementary material, S5).

To generate large 3D prints from consecutive thin layers of build material, 3D printing relies on very precise deposition of material by the printer head. Therefore, the production of accurate models is an intrinsic feature of 3D printing. McMenamin *et al.* [14] found a percentage error of 17.92% (variance 1.52%), 14.52% (variance 8.58%) and 1.29% (variance 0.02%) for printed models below 4 mm, between 4 mm and 10 mm and above 10 mm in size, respectively, using a high-end 3D printer (Z650 printer, 3D Systems, Rock Hill, SC) [10]. To test the most inexpensive and least precise printer applied in this study, the Makerbot Replicator 2 Desktop 3D Printer (Stratasys, New York, NY, USA), we compared the true XY sizes (mean of X and Y dimensions, representing the building plane) and Z sizes (representing the slice/layer direction) of the 3D printed scale cubes produced for all anatomical prints with the theoretical sizes (electronic supplementary material, S6). In overall the 3D printed scale, cubes were significantly larger than the theoretical size in both the XY- and Z-directions (*p*=0.007 for the XY-directions and *p*=0.023 for Z-direction). However, the mean error and standard deviation in the XY-direction were merely 119.00±66.84 μ*m* for printed models below 4 mm in size (a percentage error of 5.28%, variance 0.20%), 93.3±12.58 μm for printed models between 4 and 10 mm in size (a percentage error of 1.32%, variance 0.0022%), and 3.3±29.10 μm for printed models above 10 mm in size (a percentage error of 0.013%, variance 0.0034%). In the Z-direction, the mean error and standard deviation were 44.00±98.13 μm for printed models below 4 mm in size (a percentage error of 1.95%, variance 0.43%), 123.30±32.15 μm for printed models between 4 and 10 mm in size (a percentage error of 1.74%, variance 0.015%), and 70.00±52.53 μm for printed models above 10 mm in size (a percentage error of 0.28%, variance 0.011%). The error of the scale cube prints were not significantly affected by the original model size in the Z-direction (*p*=0.99) within the range of printed cube models, whereas there was a significant linear dependency between the error and original model size in the XY-direction (*p*=0.00005), and smaller prints were thus less precise than larger prints in the XY-direction (electronic supplementary material, figure S6). However, considering the size of all anatomical prints produced in this study ranging in size from 2.8 to 17.4 cm on each axis, we found that the precision of the Makerbot Replicator 2 Desktop 3D Printer and additional more precise high-end 3D printers applied in this study was sufficient to produce reliable scale models of anatomical structures of a manageable size.

As described above, alternative 3D printing technologies exist such as vat photopolymerization and directed energy deposition, allowing for the production of more fragile models. Interestingly, multi-material deposition modelling that enables production of models containing several materials has recently become available in high-end 3D printers [7]. In contrast to material extrusion and to some degree binder jetting, these technologies have still to mature in a competitive market for personal 3D printers, and may still be unachievable for small laboratories not dedicated for 3D modelling and independent research groups in comparative anatomy and physiology.

## 3. Benefits and concerns of three-dimensional printed models of anatomy and physiology

The production of physical and manageable anatomical and physiological models as described above is a natural extension to the use of virtual models widely applied in scientific papers recently [1–5]. Even though 3D virtual models represent a major leap forward compared to 2D figures, it is our belief that physical models of complex and sometimes unknown structures that can be handled much like the well-known anatomy model of the human torso, will aid in comprehending complicated features. The possibility of digitizing, electronically transferring and finally printing rare or fragile specimens is another advantage of 3D printing technology being ever-more accessible for researchers as well as private individuals. In this way, models of precious specimens can be distributed rapidly and at no risk.

An interesting property of 3D printed scale models that should be taken into account when examining printed models of originally very differently sized structures scaled to similar sizes is the concept of isometric versus allometric scaling. A 3D printed scale model has experienced a predefined degree of isometric scaling by which the linear dimensions of the object change in proportion to each other and the surface area, volume and weight change in a predictable way. However, most groups of animals show allometric changes in body proportions as well as size as they change in mass [25]. To some degree, this phenomenon can be observed when comparing the axis bone model of the European mole (figure 2J-3) and the harbour seal (figure 2K-3). Even though the models have been isometrically scaled and printed to similar sizes and generally have a similar shape, the seal axis bone is more robust than the mole equivalent, which is probably the result of allometric scaling that is often manifested as more robust skeletons of larger animals relative to smaller species [25]. Isometric scaling and subsequent 3D printing of structures in smaller animals to the size of the equivalent structures in larger animals can in this way be applied to highlight the effect of allometric scaling in comparative anatomy.

A 3D anatomical model is only as accurate as the underlying segmentations and hence relies on the quality of the image data and finally the preparation of the original sample. The print quality is also dependent on the performance of the 3D printer, however as most affordable desktop printers already provide *a*<100 μm precision, this concern only affects very small or extremely precise models. In those situations, online 3D print services exist such as http://www.shapeways.com/, http://i.materialise.com/ and http://www.sculpteo.com/en/ that allow for the production of very precise models in a wide range of materials at affordable prices.

At present, a major limiting factor of low-cost 3D printers is the lack of option to produce multi-material models. Anatomical and physiological models would benefit greatly from the option to apply several materials to produce models containing semi-transparent segments as well as segments that feel different when handled. Printed models are generally prepared in sturdy materials which prolong durability; however, this is incompatible with dissectible models that could aid teaching. Preparing anatomical models in materials similar to tissue would allow for thorough dissections that could alleviate, but by no means replace, the need for cadavers in teaching. A specialized type of 3D printing termed 3D bioprinting that seeks to shape tissues and organs from biocompatible materials, cells and supporting components using additive manufacturing techniques is a final answer to this request. However, at present, this technology needs maturation before anatomical and more importantly functional organs can be generated by the click of a button [26].

## 4. Conclusion

Additive manufacturing or rather 3D printing has, for good reasons, attracted the public awareness in recent years as this technology has the potential to liberate the creation process of prototypes and various everyday products, e.g. smart phone covers, camera brackets, art and jewellery objects, etc. [7]. Manufacturing of 3D models has already made its way into medical science, and by providing scalable hands-on models of complex structures, we believe that this technology will benefit the fields of comparative anatomy and physiology. Just as it has become the norm in recent years to provide interactive models as the electronic supplementary material for anatomical studies, providing ready-to-3D-print files of structures described would also aid the reader. Even in a digital age, anatomy and physiology are still physical disciplines and as such are best replicated by physical models.

## 5. Material and methods

Sample preparation, image acquisition, data segmentation and printing are highly dependent on the specific case of anatomical or physiological modelling. In the following, we provide a description of how the diversity of 3D printed models applied in this communication were produced.

### 5.1 Sample preparation

All animals used in this study (table 1) were collected as *post mortem* specimens and therefore no ethical approval was required. The vascular models of the coronary architecture of the giraffe and elephant hearts (figure 4) were prepared by perfusion with a mixture of CT contrast agent (Mixobar Colon, 1 g ml^−1^; Astra Tech, Sweden) and gelatin as described previously into formaldehyde-fixated hearts [27]. The anuran outflow tract models (figure 5; electronic supplementary material, S3) were prepared using soft tissue staining of a previously perfusion fixed (formaldehyde) heart of a cane toad with a 16.6% Lugol’s iodine solution (32.7 mM I_2_ and 100 mM KI in water).

**Table 1.** Specimen overview: list of species and anatomical structures included in the study and the corresponding imaging modality and 3D printing technique used to generate the physical models. (CT, computed tomography; xCT, extremity computed tomography; μCT, micro computed tomography; MRI, magnetic resonance imaging; PLA, polylactic acid.)

species	structure	imaging	3D print technique	data accessibility
red-eared slider (*Trachemys scripta elegans*, Wied-Neuwied)	skeleton, lungs	CT	material extrusion, single printer head, white PLA	electronic supplementary material, S1 (interactive model, full body), electronic supplementary material, S2 (ready-to-3D-print model, skeleton and lungs)
school shark (*Galeorhinus galeus*, Linnaeus)	axis bone	CT	material extrusion, single printer head, white PLA	http://dx.doi.org/10.5061/dryad.8787m/7 (interactive model, head), http://dx.doi.org/10.5061/dryad.8787m/8 (ready-to-3D-print model, axis bone)
Atlantic cod (*Gadus morhua*, Linnaeus)	axis bone	xCT	material extrusion, single printer head, white PLA	http://dx.doi.org/10.5061/dryad.8787m/5 (interactive model, head), http://dx.doi.org/10.5061/dryad.8787m/6 (ready-to-3D-print model, axis bone)
African lungfish (*Protopterus annectens*, Owen)	axis bone	xCT	material extrusion, single printer head, white PLA	http://dx.doi.org/10.5061/dryad.8787m/24 (interactive model, head), http://dx.doi.org/10.5061/dryad.8787m/25 (ready-to-3D-print model, axis bone)
tiger salamander (*Ambystoma tigrinum*, Green)	axis bone, anterior surface and bone	xCT	material extrusion, single printer head, white PLA; binder jetting, single printer head, bronze infused stainless steal	http://dx.doi.org/10.5061/dryad.8787m/1 (interactive model, head), http://dx.doi.org/10.5061/dryad.8787m/2 (ready-to-3D-print model, axis bone)
European common frog (*Rana temporaria*, Linnaeus)	axis bone	μCT	material extrusion, single printer head, white PLA	http://dx.doi.org/10.5061/dryad.8787m/26 (interactive model, head), http://dx.doi.org/10.5061/dryad.8787m/27 (ready-to-3D-print model, axis bone)
bearded dragon (*Pogona*sp.)	axis bone	xCT	material extrusion, single printer head, white PLA	http://dx.doi.org/10.5061/dryad.8787m/22 (interactive model, head), http://dx.doi.org/10.5061/dryad.8787m/23 (ready-to-3D-print model, axis bone)
puff adder (*Bitis arietans*, Merrem)	axis bone	xCT	material extrusion, single printer head, white PLA	http://dx.doi.org/10.5061/dryad.8787m/3 (interactive model, head), http://dx.doi.org/10.5061/dryad.8787m/4 (ready-to-3D-print model, axis bone)
black caiman (*Melanosuchus niger*, Spix)	axis bone	CT	material extrusion, single printer head, white PLA	http://dx.doi.org/10.5061/dryad.8787m/15 (interactive model, head), http://dx.doi.org/10.5061/dryad.8787m/16 (ready-to-3D-print model, axis bone)
ostrich (*Struthio camelus*, Linnaeus)	axis bone	CT	material extrusion, single printer head, white PLA	http://dx.doi.org/10.5061/dryad.8787m/31 (interactive model, head), http://dx.doi.org/10.5061/dryad.8787m/32 (ready-to-3D-print model, axis bone)
European mole (*Talpa europaea*, Linnaeus)	axis bone	xCT	material extrusion, single printer head, white PLA	http://dx.doi.org/10.5061/dryad.8787m/33 (interactive model, head), http://dx.doi.org/10.5061/dryad.8787m/34 (ready-to-3D-print model, axis bone)
harbour seal (*Phoca vitulina*, Linnaeus)	axis bone	CT	material extrusion, single printer head, white PLA	http://dx.doi.org/10.5061/dryad.8787m/17 (interactive model, head), http://dx.doi.org/10.5061/dryad.8787m/18 (ready-to-3D-print model, axis bone)
giraffe (*Giraffa camelopardalis*, Linnaeus)	axis bone, coronary arteries	CT	material extrusion, single printer head, white PLA (axis bone); material extrusion, dual printer head, ivory ABS (coronary arteries)	http://dx.doi.org/10.5061/dryad.8787m/9 (interactive model, head), http://dx.doi.org/10.5061/dryad.8787m/10 (ready-to-3D-print model, axis bone), http://dx.doi.org/10.5061/dryad.8787m/11 (interactive model, heart), http://dx.doi.org/10.5061/dryad.8787m/12 (ready-to-3D-print model, coronary arteries)
harbour porpoise (*Phocoena phocoena*, Linnaeus)	lungs, internal skull surface	CT, MRI	material extrusion, single printer head, red PLA (internal skull surface; material extrusion, dual printer head, ivory ABS (lungs)	http://dx.doi.org/10.5061/dryad.8787m/19 (interactive model, full body), http://dx.doi.org/10.5061/dryad.8787m/20 (ready-to-3D-print model, internal skull surface), http://dx.doi.org/10.5061/dryad.8787m/21 (ready-to-3D-print model, lungs)
African bush elephant (*Loxodonta Africana*, Blumenbach)	coronary arteries	CT	material extrusion, dual printer head, ivory ABS	http://dx.doi.org/10.5061/dryad.8787m/13 (interactive model, heart), http://dx.doi.org/10.5061/dryad.8787m/14 (ready-to-3D-print model, coronary arteries)
cane toad (*Rhinella marina*, Linnaeus)	conus arteriosus, truncus arteriosus	μCT	material extrusion, single printer head, golden PLA; binder jetting, single printer head, full colour sandstone	http://dx.doi.org/10.5061/dryad.8787m/28 (interactive model, heart), http://dx.doi.org/10.5061/dryad.8787m/29 (ready-to-3D-print model, conus), http://dx.doi.org/10.5061/dryad.8787m/30 (ready-to-3D-print model, truncus)
Hadrosaur (*Hadrosaurus* sp., Leidy)	lower femur fraction	photographic	binder jetting, single printer head, full colour sandstone	for interactive model and a complete examination of the fossil, see [23]

### 5.2 Image acquisition

Data obtained to generate the models in figures 1 and 2, the lung model in figures 3–5, electronic supplementary material, S1–S3 and S5 were acquired using three separate CT systems. For large samples (red-eared slider, shark, caiman, ostrich, seal, porpoise lungs, giraffe axis and heart and elephant heart), low-resolution scans were performed on a clinical CT system (Siemens Somatom; Siemens Medical Solutions, Forchheim, Germany) with the following parameters: field-of-view depending on sample size; 0.6×0.6×0.6 mm^3^ voxel size; 100 kVp tube voltage; 260 μAs tube charge, resulting in an acquisition time of 5–20 s depending on sample size. For medium samples (cod, lungfish, salamander, bearded dragon, puff adder and mole), medium resolution scans were performed on a clinical extremity CT system (Scanco Medical XtremeCT; Scanco, Brüttisellen, Switzerland) with the following parameters: field-of-view depending on sample size; 0.082×0.082×0.082 mm^3^ voxel size; 59.4 kVp tube voltage; 119 μAs tube charge, resulting in an acquisition time of 35–60 min depending on sample size. For small samples (frog and cane toad), high-resolution scans were performed on a specimen CT scanner (Scanco Medical μCT-35; Scanco) with the following parameters: field-of-view depending on sample size; 0.015×0.015×0.015 mm^3^ voxel size; 55 kVp tube voltage; 116 μAs tube charge, resulting in an acquisition time of approximately 10 h.

MRI data obtained to generate the model of the internal surface of the harbour porpoise skull were acquired using a clinical 1.5 T system (Magnetom Avanto, Siemens Medical Solutions, Forchheim, Germany) with the following parameters: ultrafast gradient-echo sequence; 31.2×38.4 cm^2^ field-of-view; 1×1×1 mm^3^ voxel size; TR: 14.8 ms; TE: 3.38 ms; *θ*: 15^°^; three numbers of averages, resulting in an acquisition time of 2 h.

Photos used to generate the full colour digital and printable model of the hadrosaur femur were captured using a Nikon D700 SLR camera generating images of a 300 dpi resolution and a 24 bit colour depth. Autodesk 123D Catch (Autodesk Inc.) was used to convert the 67 overlapping photos into a 3D digital model using standard settings.

### 5.3 Data segmentation

Amira 5.6 (FEI, Visualization Sciences Group) was used for data segmentation, and the LabelField module allowed for manual creation of polygon meshes (surface models). Segmentation of bone structures and contrast agent-filled vasculature from CT data was performed semi-automatically selecting a seeding point and growing the region of interest using the Magic Wand tool of the LabelField module selecting neighbouring voxels with Hounsfield values above 700 for bone and above 300 for contrast-filled blood vessels. MRI and μCT imaging of soft tissue-stained samples generates data in which the interpretation of pixel values is less straightforward and highly set-up dependent compared to CT imaging. Segmentation of anatomical structures from MRI and μCT using soft tissue staining was performed manually using image contrast differences and knowledge of anatomical architecture. Following segmentation, the SurfaceGen module with an activated constrained smoothing setting was applied to generate surface-rendered models that were saved in the Wavefront format (.obj). Interactive PDF files of each model were generated [3], and images for figures 1–5 were exported from these documents. In Netfabb basic, all print models were isometrically rescaled to fit the build volume of the 3D printer, and for axis prints in figure 2 to become similar sized. Rescaled surface models were exported in STL-format for monochromatic models, VRML-format for artificially coloured models (cane toad outflow tract) and X3D for true colour models (hadrosaur fossil).

### 5.4 Printing

All printed models made in PLA plastic in this study were produced with a Makerbot Replicator 2 Desktop (Stratasys) material extrusion 3D printer with a single printer head having a theoretical layer (Z) resolution of 0.10 mm. Standard settings were used. To test the precision of printing, 3D printed cubes additionally used as scale cubes for individual models (2.5×2.5×2.5 mm^3^ scaled equivalent to each anatomical and physiological model) were measured in the Z-direction and the XY-direction (mean of X- and Y-dimensions) with an electronic vernier calliper and compared to theoretical sizes using standard statistics (Student’s *t*-test and linear regression). To produce complex models of coronary vasculature and pulmonary structures, a uPrint SE (Stratasys) material extrusion 3D printer with a dual printer head using ABS (model material) and SR-30 (soluble support material) having a layer resolution of 0.254 mm was used with standard settings. To produce full colour sandstone and metal 3D prints, we applied the commercial online platform from Shapeways 3D Printing Service and Marketplace (https://www.shapeways.com/). Both full colour sandstone and metal prints were produced using binder jetting technology.

## Supplementary Material

Supplementary material 1. Three-dimensional interactive model of red-eared slider. Three-dimensional rendered interactive model generated from x-ray computed tomography imaging of the red-eared slider (Trachemys scripta elegans) used in Figure 1.The interactive PDF file should be viewed in Adobe Acrobat Reader 9 or higher. To activate the 3D feature click the model. Using the cursor it is now possible to rotate, zoom, pan the model and in the model tree all segments of the model can be turned on/off or made transparent.

## Supplementary Material

Supplementary material 2. Ready-to-3D-print model of red-eared slider. Ready-to-3D-print STL-file of the red-eared slider (Trachemys scripta elegans) used in Figure 1. The STL file can be imported directly into the software of a 3D printer and printed. The model was scaled (65%) to fit the printer bed of a Makerbot Replicator 2 Desktop 3D Printer (Stratasys, New York, USA) (28.5ͯ15.3ͯ15.5 cm3), however it can be rescaled in printer software to any desired size.

## Supplementary Material

Supplementary material 5. 3D printing of anatomical structures in metal. Three-dimensional interactive model of the anterior part of a tiger salamander (Ambystoma tigrinum) separated into a surface reconstruction (right side of animal) and a skeleton reconstruction (left side of animal). The original 3D model prepared using μCT imaging is displayed next to a redigitised model of a 3D print produced in bronze infused stainless steel. To activate the 3D feature click the model. Using the cursor it is now possible to rotate, zoom, pan the model and in the model tree all segments of the model can be turned on/off or made transparent.

## Supplementary Material

Supplementary material 6. Precision of 3D printed structures. Bland–Altman plot (difference (dif.) over average (avg.)) comparing the theoretical width in the Z-direction (slice/layer) and XY-direction (mean of X and Y dimension) to the physical width of 3D printed cubes used as scale cubes in preceding figures. The printed scale cubes are generally slightly larger than the theoretical size and the XY precision decreases in smaller prints.

## Supplementary Material

Supplementary_material

## Data accessibility

Interactive models and ready-to-3D-print files are available from Dryad Digital Repository (http://dx.doi.org/10.5061/dryad.8787m). The interactive PDF files should be viewed in Adobe Acrobat Reader 9 or higher. To activate the 3D feature click the model. Using the cursor, it is now possible to rotate, zoom, pan the model and in the model tree all segments of the model can be turned on/off or made transparent.
