# Supplementary figures and images for: From tissue to silicon to plastic: three-dimensional printing in comparative anatomy and physiology
Source: R Soc Open Sci. 2016 Mar 2;3(3):150643. doi: 10.1098/rsos.150643 (PMC4821264; doi:10.1098/rsos.150643)

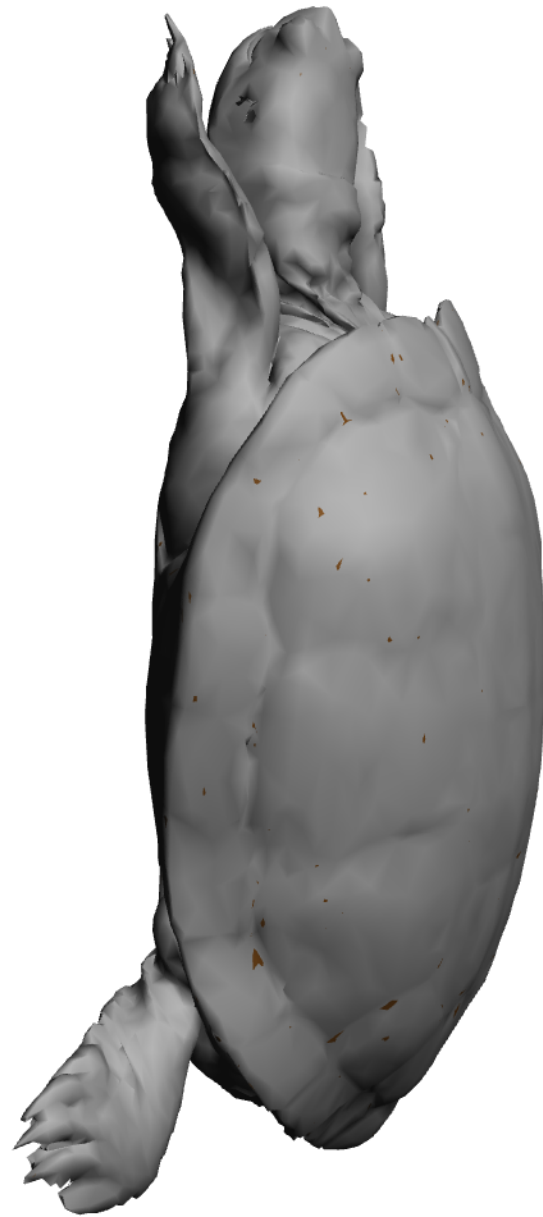

Supplement: Supplementary material 1. Three-dimensional interactive model of red-eared slider. Three-dimensional rendered interactive model generated from x-ray computed tomography imaging of the red-eared slider (Trachemys scripta elegans) used in Figure 1.The interactive PDF file should be viewed in Adobe Acr [file rsos150643supp1.pdf]

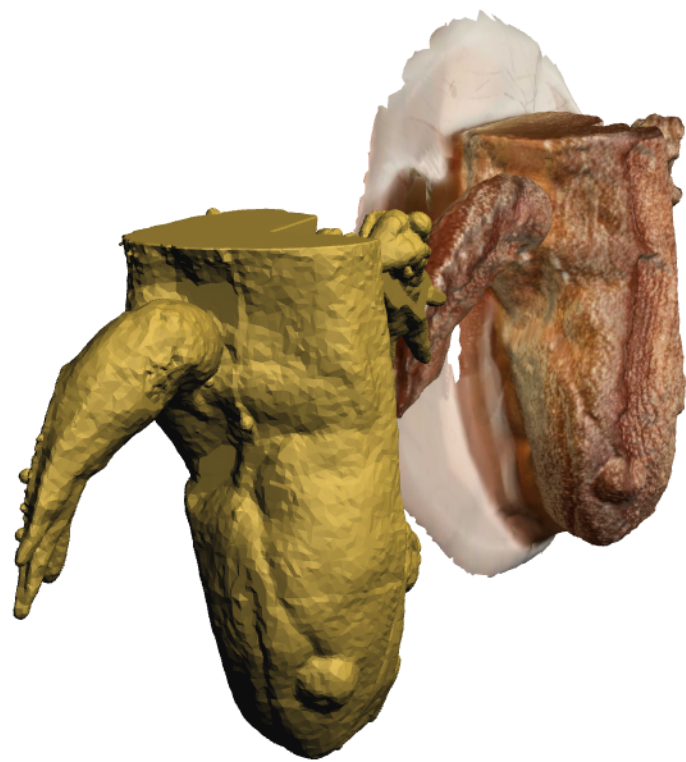

Supplement: Supplementary material 5. 3D printing of anatomical structures in metal. Three-dimensional interactive model of the anterior part of a tiger salamander (Ambystoma tigrinum) separated into a surface reconstruction (right side of animal) and a skeleton reconstruction (left side of animal). The origina [file rsos150643supp3.pdf]

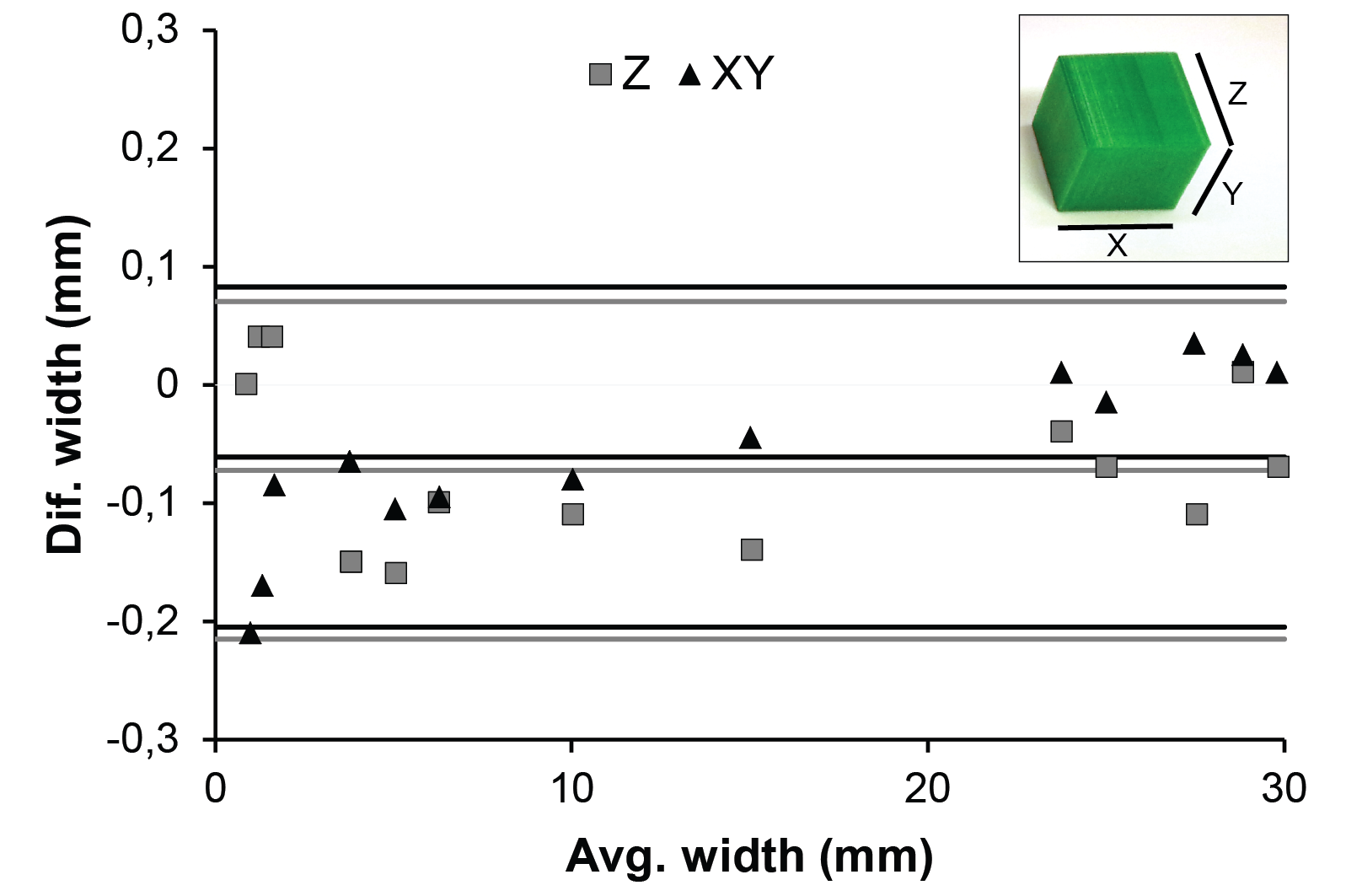

Supplement: Supplementary material 6. Precision of 3D printed structures. Bland–Altman plot (difference (dif.) over average (avg.)) comparing the theoretical width in the Z-direction (slice/layer) and XY-direction (mean of X and Y dimension) to the physical width of 3D printed cubes used as scale cubes in prece [file rsos150643supp4.tif]

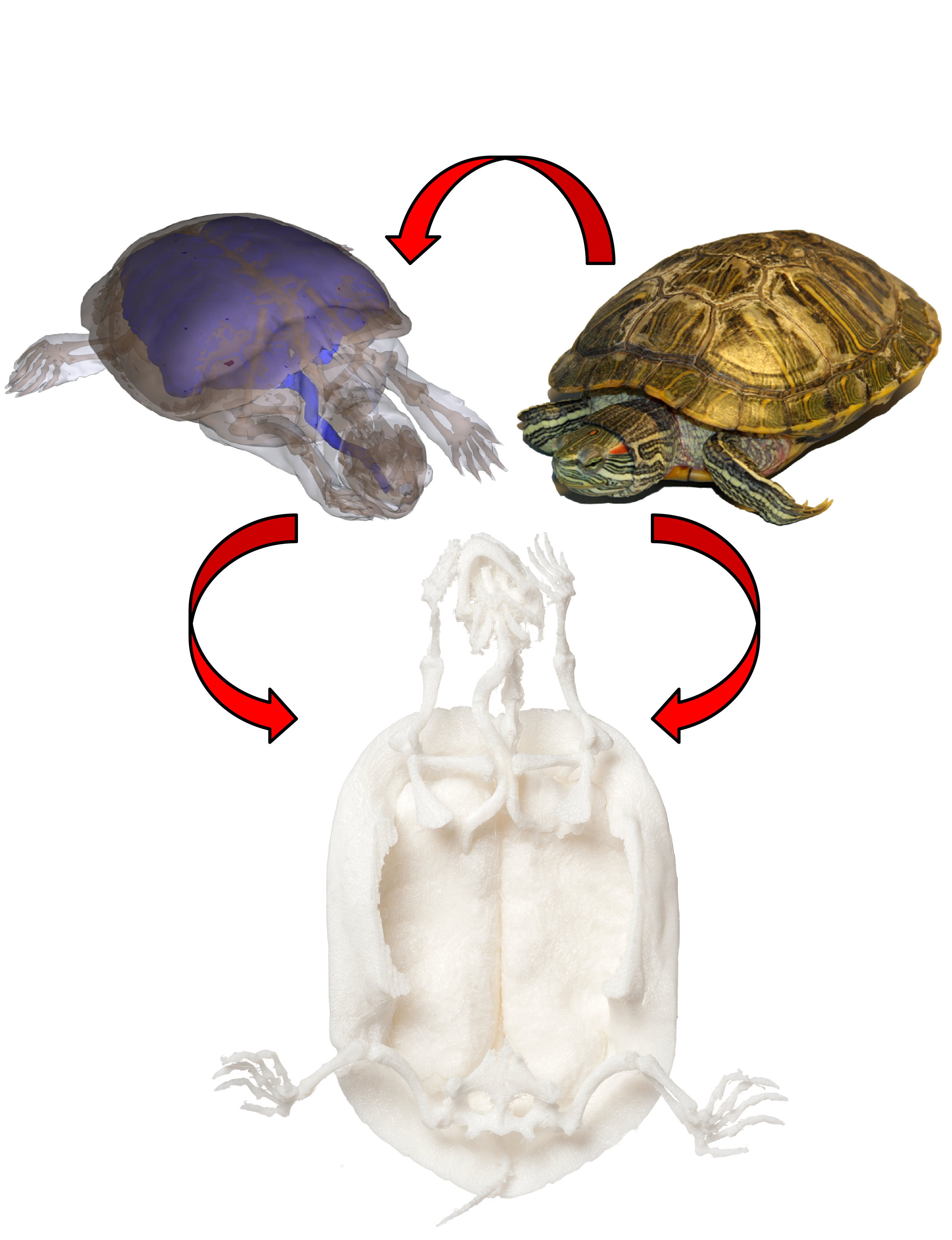

Supplement: Supplementary_material [file rsos150643supp5.tif]
